# A test prototype of a novel flexible video laryngoscope and preliminary verification in a difficult airway management simulator

**DOI:** 10.1186/s12938-022-01043-1

**Published:** 2022-10-03

**Authors:** Fei Xu, Chang Liu, Yang Zhou, Min Li, Xiangyang Guo

**Affiliations:** Department of Anesthesiology, Peking University Third Hospital, Beijing, 100191 China

**Keywords:** Difficult airway management simulator, Flexible video laryngoscope, Intubation, Video laryngoscope

## Abstract

**Background:**

To verify a test prototype of a novel flexible video laryngoscope in a difficult airway management simulator and to compare the efficacy of the flexible video laryngoscope with that of a conventional video laryngoscope.

**Methods:**

Fifteen clinical anesthesiologists performed endotracheal intubation with a flexible video laryngoscope and a conventional video laryngoscope in a difficult airway management simulator in the neutral position with intermediate and difficult mouth opening. The rate of intubation success, intubation time, and classification of glottic exposure were recorded. After endotracheal intubation, participants were asked to assess the difficulty of intubation of the two laryngoscopes.

**Results:**

The success rate of endotracheal intubation with flexible video laryngoscope was significantly higher than that with video laryngoscope in neutral positions with both intermediate (*P* = 0.025) and difficult (*P* = 0.005) mouth opening. The Cormack Lehane score of the flexible video laryngoscope was significantly lower than that of the video laryngoscope in the neutral position with intermediate mouth opening (*P* < 0.001) and difficult mouth opening (*P* < 0.001). There was no significant difference in intubation time in the neutral position with intermediate mouth opening (*P* = 0.460) or difficult mouth opening (*P* = 0.078). The difficulty score of endotracheal intubations with the flexible video laryngoscope was also significantly lower than that of the video laryngoscope in the neutral position with intermediate mouth opening (*P* = 0.001) and difficult mouth opening (*P* = 0.001).

**Conclusions:**

Compared with conventional video laryngoscopy, flexible video laryngoscopy can provide superior glottic exposure and improve the success rate of intubation in a difficult airway management simulator.

**Supplementary Information:**

The online version contains supplementary material available at 10.1186/s12938-022-01043-1.

## Introduction

Endotracheal intubation of patients with difficult airways has always been one of the most challenging tasks in clinical practice. Failed intubation can lead to airway injury and even death [1–3]. Previous studies have shown that the incidence of difficult endotracheal intubation ranges from 1% to 11.3% [4–7], and thus far, no perfect device has been designed to address this scenario. Therefore, an emergency airway (cannot intubate, cannot ventilate), which occurs from time to time, often endangers the patient’s life and brings severe challenges to airway management. Therefore, we designed a new laryngoscope to overcome the shortcomings of the current intubation tools and to meet the needs of the clinic.

## Background

Current intubation tools mainly include direct laryngoscopes, visual laryngoscopes, visual rigid laryngoscopes, and flexible bronchoscopes. Among these intubation tools, the direct laryngoscope has long been the most commonly used, but it has inherent limitations because it requires a direct line of sight between the operator’s eyes and the glottis [8], which means that when a direct laryngoscope is used, the intubation conditions of patients are required to be higher; if these conditions cannot be met, the rate of intubation failure increases. Therefore, the direct laryngoscope is not suitable for difficult airways.

As an alternative to direct laryngoscope, video laryngoscope has developed rapidly in recent years. Video laryngoscopy relies on digital technology to transmit an image from the tip of the laryngoscope to a monitor, so it does not require a direct line of sight between the operator’s eyes and the glottis. Compared to the conventional direct laryngoscope, the video laryngoscope has a wide field of view and can clearly display the structure of the pharyngeal cavity. Previous studies have shown that visual laryngoscopy has a higher intubation success rate than direct laryngoscopy [9–11]. At present, there are various visual laryngoscopes with different shapes, styles and sizes available on the market, mainly including VividTrac, Airway Scope, Airtraq, C Mac, King Vision, etc. [9–13]. However, the shape of the video laryngoscope blade is fixed. Sometimes it is difficult to insert the video laryngoscope into the mouth cavity or expose the glottis in some patients with limited mouth opening, limited neck movement, etc., which results in intubation failure.

Another type of laryngoscope is the visual rigid laryngoscope, which mainly includes the Trachway video intubating stylet, Levitan FPS optical stylet, MultiViewScope, Optiscope video stylet, etc. [14–18]. As a tool for guiding intubation in endotracheal tubes, the visual field is relatively small, and sometimes it is difficult to identify the structure of the pharyngeal cavity. In addition, the device has no suction function, and the lens can easily be obscured by secretions.

As the distal end of the insertion part of the flexible bronchoscope can be flexibly changed to find the glottis by adjusting the control lever on the laryngoscope handle, it is a well-recognized airway management tool used in patients with difficult airways. However, the flexible bronchoscope is also a device for guiding intubation with endotracheal tubes, the insertion part of the flexible bronchoscope is thin, and the field of vision is confined. Sometimes it is difficult for the operator to distinguish the structure of the pharyngeal cavity. In addition, the suction function of the flexible bronchoscope is usually not efficient, and the lens is easily obscured by secretions or blood, resulting in blurred vision. Given these facts, a novel device that can ensure the efficacy of intubation in difficult airways is needed.

As we have analyzed above, the previous intubation tools either have a wide field of view but are not flexible or are flexible but have a small field of view. Here, we introduce a novel difficult airway intubation tool designed and manufactured by us. This device keeps the basic structure of the flexible bronchoscope by making the flexible bronchoscope thicker and shorter, thus retaining its flexibility. A protrusion is added at the front end of the lens to increase its field of vision, thus inheriting the advantages of the visual laryngoscope with a wide field of view. Therefore, it combines the advantages of the "wide field of view" of the video laryngoscope and the "flexibility" of the flexible bronchoscope. At the same time, to enhance its suction function, the diameter of the insertion part and the suction channel were increased (their current diameters are 12 mm and 3 mm, respectively.). As this modified laryngoscope can no longer guide intubation in endotracheal tubes like a flexible bronchoscope does, we installed an endotracheal tube guide device on one side of the laryngoscope. Through these reformations, the advantages of the previous intubation tools are retained, and their disadvantages are overcome.

Currently, we have created the device as a test prototype and have named it a “flexible video laryngoscope (FVL)”. To further improve this instrument and prepare for clinical verification in the future, we carried out this preliminary study to verify the efficacy of flexible video laryngoscopy in difficult airway scenarios.

## Results

A total of 15 participants were enrolled in this study. The details of the participant characteristics, such as sex, age, experience in anesthesia, experience in using a video laryngoscope and experience in using a flexible bronchoscope to perform intubation of difficult airway scenarios, are described in Table 1.

**Table 1:** Participant characteristics

Characteristic	Description
Sex (male/female)	6/9
Age (years)	40.13 ± 5.32
Experience in anesthesia (years, median (min–max))	11 (5–25)
Experience in using a video laryngoscope	
100–500 times (*n*)	4
More than 500 times (*n*)	11
Experience in using a flexible bronchoscope to perform intubations in a difficult airway scenario	
Less than 10 times (*n*)	5
10–50 times (*n*)	6
More than 50 times (*n*)	4

The success rate of endotracheal intubation with the FVL was significantly higher than that with a video laryngoscope, regardless of whether the patient was in a neutral position with intermediate mouth opening (*P* = 0.025, 100% vs. 66.7%) or with difficult mouth opening (*P* = 0.005, 100% vs. 46.7%).

The Cormack Lehane score of the FVL was significantly lower than that of the video laryngoscope in the neutral position with intermediate mouth opening (*P* < 0.001) and difficult mouth opening (*P* < 0.001). There was no significant difference in intubation time in the neutral position with intermediate mouth opening (*P* = 0.460) or difficult mouth opening (*P* = 0.078). The difficulty score of endotracheal intubations with the FVL was also significantly lower than that of video laryngoscopy in the neutral position with intermediate mouth opening (*P* = 0.001) and difficult mouth opening (*P* = 0.001), as shown in Table 2.

**Table 2:** Comparison of the indices between flexible video laryngoscope and visual laryngoscope

Index	Neutral position with intermediate mouth opening			Neutral position with difficult mouth opening		
	FVL	VL	*P*	FVL	VL	*P*
Cormack Lehane score	1 (1–1)	2 (2–3)	< 0.001	1 (1–1)	3 (3–3)	< 0.001
Intubation time(s)	40 (30–48)	27 (18–90)	0.460	33 (28–43)	90 (21–90)	0.078
Score of difficult intubations	1 (1–4)	5 (3–7)	0.001	1 (1–3)	8 (8–10)	0.001

Data are median (interquartile range). FVL: flexible video laryngoscope; VL: video laryngoscope

## Discussion

This study showed that the new flexible video laryngoscope (FVL) performs well in a difficult airway management simulator. All glottic exposures were grade 1, and the success rates for intubation were 100%, which were much higher than those of video laryngoscopy. The difficulty score of intubations with the FVL was significantly lower than that with the video laryngoscope. Although the intubation success rate of the video laryngoscope reached 46.7% in the neutral position with difficult mouth opening, the glottic exposure could only reach grade 3 (only the epiglottis but no portion of the glottis was visible). All tracheal tubes were blindly inserted into the glottis.

At present, the main intubation tools used to address difficult airways are video laryngoscopy and flexible bronchoscopy. Video laryngoscopy has played an increasingly important role in airway management. Although there are many kinds of video laryngoscopes on the market, they mainly improve the exposure of the glottis by changing the shape and angle of the laryngoscope blade. Considering that sometimes the larger angulated blade shape cannot be inserted into the mouth of patients with their head in the neutral position and difficult mouth opening, we did not choose the larger angulated blade shape in this study. In addition, the visual laryngoscope we selected is most widely used in China.

Because the insertion part of the flexible bronchoscope is flexible, it is suitable for various types of difficult airways. However, its disadvantages are also very prominent: it needs enough space in front of the lens; otherwise, the visual field will be small, and sometimes it is also difficult for the operator to distinguish the structure of the pharyngeal cavity, especially for those who are not skilled enough. In addition, the suction channel is slender, the suction function is poor, and the lens is easily obscured by secretions or blood.

This new design inherits the basic structure and function of flexible bronchoscopy, that is, it retains its flexibility. On this basis, its insertion becomes thicker and shorter; consequently, the suction channel can be enlarged to improve its suction function, and the secretions in the oropharyngeal cavity can be removed rapidly to avoid lens contamination. After induction of anesthesia, induction agents and neuromuscular blocking drugs reduce the upper airway muscle tone, resulting in upper airway narrowing and obstruction. By adding a protrusion at the front end of the insertion part, the collapsed pharyngeal cavity tissue can be lifted, so the space of the pharyngeal cavity expands, which is helpful for the operator to visualize the structure of the pharyngeal cavity. Because the lifting force of the front end of the insertion part is not very strong, the protrusion cannot lift the epiglottis from the epiglottic valley in this difficult airway management simulator; it needs to go underneath the epiglottis and lift the epiglottis to expose the glottis.

Since the insertion part of the FVL is thick and equipped with a protrusion at the front end of the insertion part, which makes it easier to expose the glottis, it cannot be placed inside the ETT as a conventional flexible bronchoscope that can be inserted into the trachea together with the ETT. We inserted the ETT by a guiding tunnel beside the insertion part and a matched guiding tube and stylet. This method changes the traditional idea of guidance within the ETT, which eliminates the ETT’s constraints on the insertion part’s diameter.

Both the stylet and the guiding tube have certain flexibility. The reason why we use the stylet to guide the guiding tube is to make the guiding tunnel as slender as possible, so the insertion part will not occupy too much space in the pharyngeal cavity. Of course, the diameter of the guiding channel can be appropriately increased, so that the guiding tube can enter the glottis directly through the guiding channel. In this way, the operation of the stylet can be omitted, and the steps are reduced.

In this study, we used a reinforced endotracheal tube. In the head neutral position, the endotracheal tube has a large curvature (approximately 90 degrees) when passing through the oral cavity to the posterior pharyngeal wall. The reinforced endotracheal tube is softer, and the shape is easier to change. It is easier to pass through the oropharyngeal cavity when sliding along the stylet.

In addition, the FVL has a ventilation channel, which can be connected to a high-flow oxygen source during intubation or to a high-frequency ventilator for high-frequency ventilation during intubation.

In this study, we did not compare this design with flexible bronchoscopy because the advantages of the FVL cannot be fully verified in a difficult airway management simulator. For instance, the function of suction and the enlargement of visualization of pharyngeal cavity needs to be verified clinically.

We want to emphasize that this FVL is only a prototype, and some details have not been completely finalized. Although this difficult airway management simulator can better simulate the situation of difficult airways by changing the mouth opening and neck movement and reliably verify the performance of the FVL in difficult airways, the real situation in the clinic is much more complicated. For example, the narrowing and obstruction of the upper airway after the induction of anesthesia, the interference of secretions on the lens, etc., all need to be further verified clinically.

## Conclusions

This study showed that the new flexible video laryngoscope performs well in a difficult airway management simulator. All glottic exposures were grade 1, and the success rate of intubation was 100%, which was much higher than that of video laryngoscopy. Moreover, the difficulty score of FVL intubation was significantly lower than that of video laryngoscopy.

## Proposed methods

The local ethics committee (Peking University Third Hospital, Beijing, China) determined that the verification of this novel flexible video laryngoscope and video laryngoscope in the difficult airway management simulator did not require ethical approval. Fifteen anesthesiologists who had worked for more than 5 years were enrolled in the study with written consent for the anonymous use of their data and completed a questionnaire about their personal information and experience in airway management.

A difficult airway management simulator (MW14, Kyoto Kagaku Co. Ltd, Kyoto, Japan. Certification: ISO 9001:2015, ISO 14001:2015; Fig. 1) was used in this study. The head extension can be adjusted to different angles, and the mouth opening can be adjusted within three different widths to simulate different levels of difficult airway. (Easy: mouth opening of approximately 5 cm; intermediate: mouth opening of approximately 3 cm; difficult: mouth opening of approximately 1.5 cm). In this study, the neck extension angle was adjusted to a neutral position, and the mouth opening was intermediate and difficult (simulating the situation of difficult airway).

**Fig. 1:**
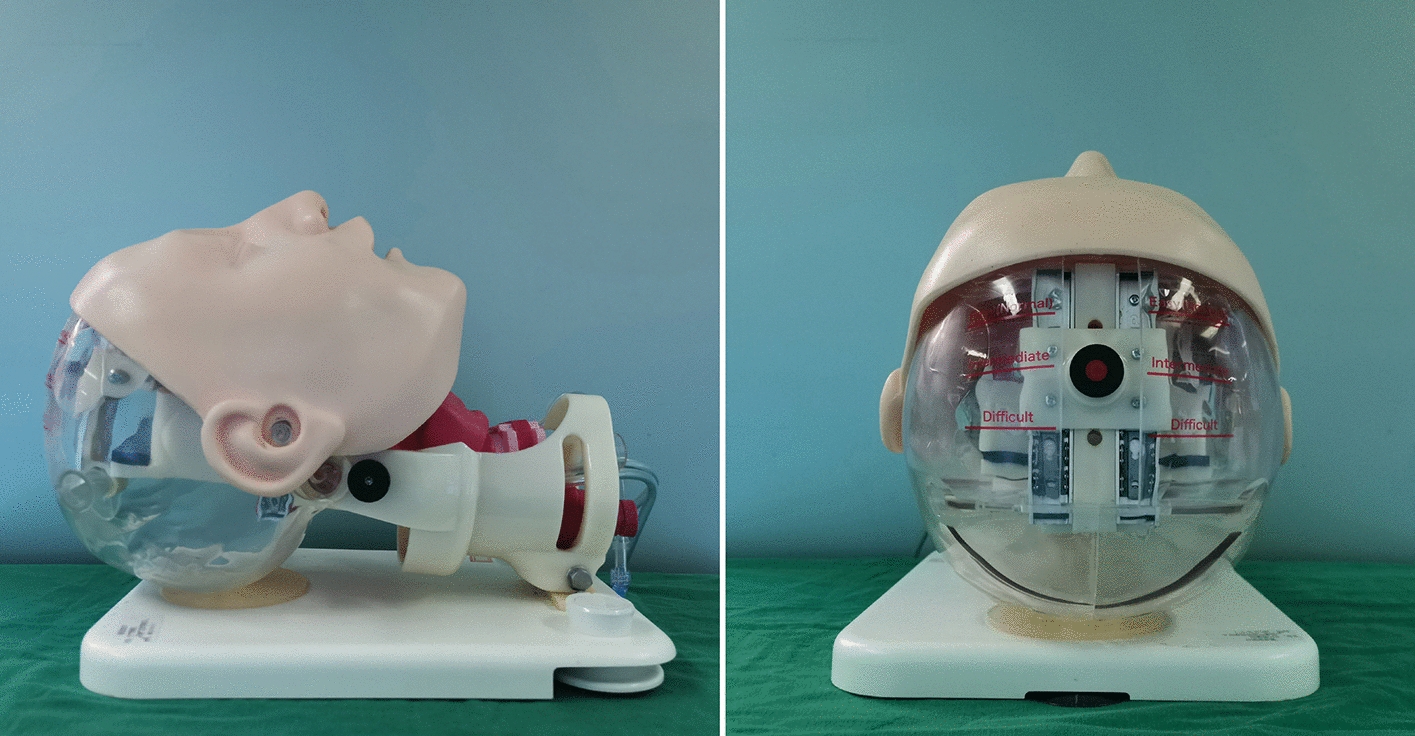
Difficult airway management simulator

**Fig. 2:**
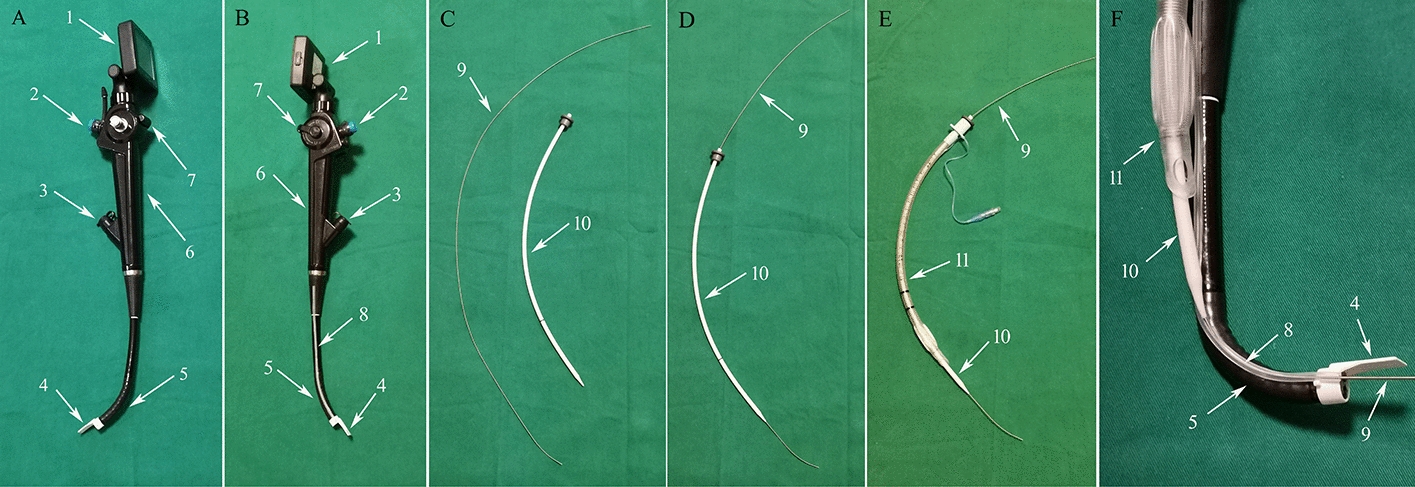
Composition and structure of the flexible video laryngoscope. The structure of the flexible video laryngoscope (**A**–**C**); the guiding tube passaged along the stylet (**D**); the endotracheal tube fixed outside the guiding tube (**E**); when the guiding tube slides along the stylet, the stylet is pulled apart from the guiding channel together with the guiding tube and endotracheal tube (**F**). 1. Display screen; 2. Suction port; 3. Ventilation port; 4. Protrusion; 5 Bending part; 6. Laryngoscope handle; 7. Control lever; 8. Guiding channel; 9. Stylet; 10. Guiding tube

The structure of the flexible video laryngoscope (Fig. 2A–C): the FVL includes three parts: a display screen in the proximal part (1), a laryngoscope handle in the middle (6) and a distal insertion part. The handle is equipped with a control lever (7), a suction port (2) and a ventilation port (3). At the distal aspect of the insertion part is a bending section. (5), which is controlled by the control lever. A protrusion (4) is installed at the distal tip of the insertion part, and the movement direction of the protrusion is the same as that of the bending section. A guiding channel (8) is located on one side of the insertion part with a potential space on the channel wall. The FVL is also equipped with a stylet (9) and a guiding tube (10) (Fig. 2C). The guiding tube can be passed along the stylet (Fig. 2D), and the endotracheal tube (ETT) can be fixed outside the guiding tube (Fig. 2E). The stylet can pass the glottis through the guiding channel, and as the guiding tube moves forward along the stylet, it can be pulled apart from the guiding channel together with the guiding tube and ETT (Fig. 2F).

**Fig. 3:**
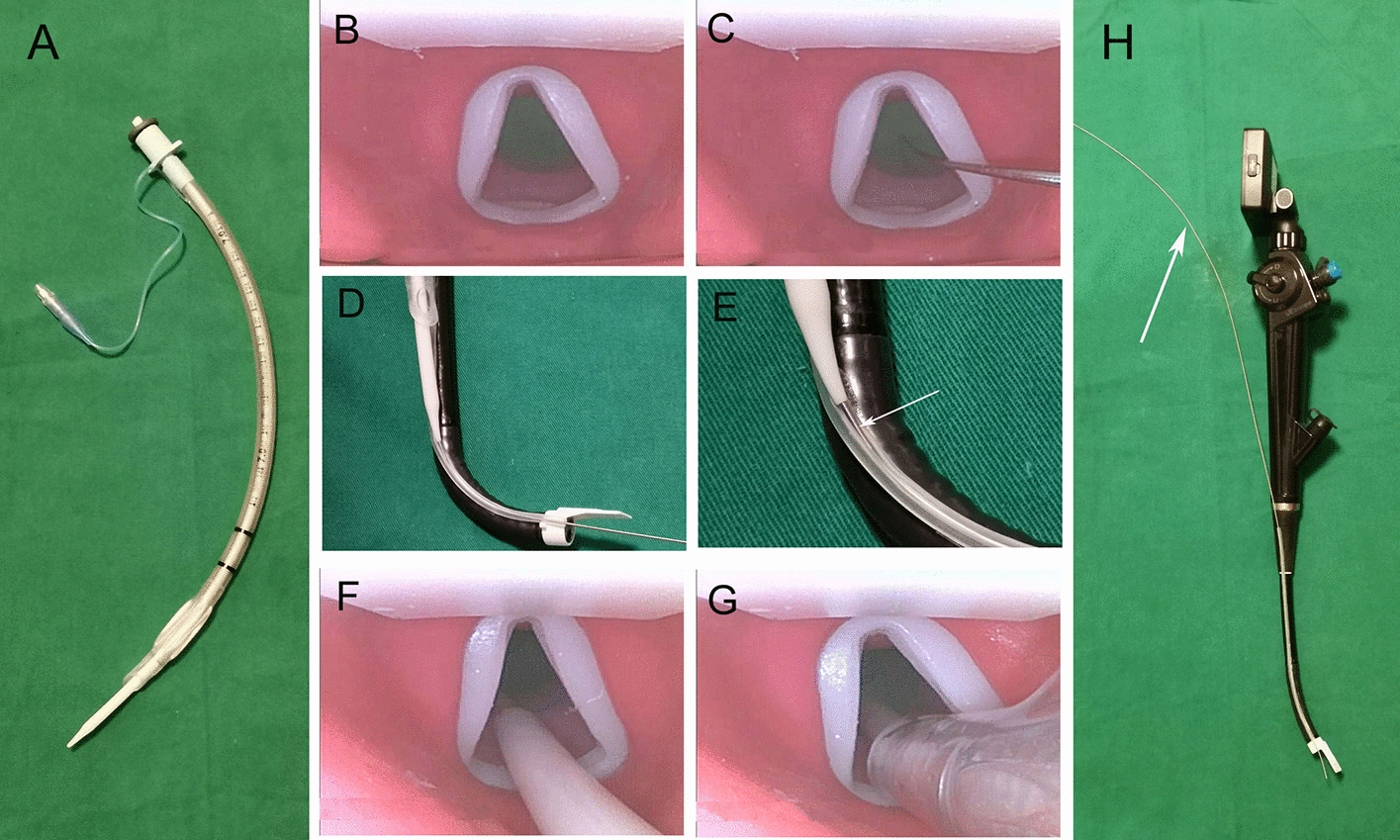
Application of the flexible video laryngoscope. The endotracheal tube is fixed outside the guiding tube (**A**); the exposed glottis is observed (**B**); the stylet is inserted into the glottis (**C**); the guiding tube together with the endotracheal tube slides along the stylet (**D**); the stylet is pulled apart from the guiding channel together with the guiding tube and endotracheal tube (**E**); the arrow indicates the stylet; the guiding tube (**F**) and endotracheal tube (**G**) is pushed into the glottis; the front part of the stylet is installed into the guiding tunnel before inserting the laryngoscope (H); and the arrow indicates the stylet

Application of the FVL: the operator installed the guiding tube into the ETT in advance (Fig. 3A) and then inserted the insertion part of the FVL into the oral cavity. As the FVL continued moving forward past the hard palate, the soft palate, and the uvula and then into the pharynx, the operator could adjust the tip of the FVL by pressing the control lever to lift the obstruction tissue. When the epiglottis was visualized, the tip of the FVL was moved forward, and then the control lever was pressed to tilt the protrusion underneath the epiglottis. Then, the epiglottis was lifted to expose the glottis (Fig. 3B). After exposing the glottis, the operator inserted the stylet into the glottis along the guide channel (Fig. 3C) and then let the assistant install the guiding tube with the ETT through the proximal end of the stylet. Then, the ETT (together with the guiding tube) was pushed forward along the stylet (Fig. 3D) into the trachea. As the guiding tube moves forward, the stylet could be pulled apart from the guiding channel together with the guiding tube and ETT (Fig. 3E). The process of pushing the guiding tube and ETT into the glottis was monitored by the operator from the display screen (Fig. 3F, G). Finally, the stylet together with the guiding tube was withdrawn from the endotracheal tube, and the FVL was withdrawn from the oral cavity; that is, endotracheal intubation was completed (Additional file 1). The front part of the stylet could also be installed into the guiding channel before inserting the FVL (Fig. 3H), and the proximal end of the stylet could be fixed to the handle by hand. After the glottis was exposed, the stylet could be pushed forward to enter the trachea. In this study, the front part of the stylet was inserted into the guiding channel in advance.

**Fig. 4:**
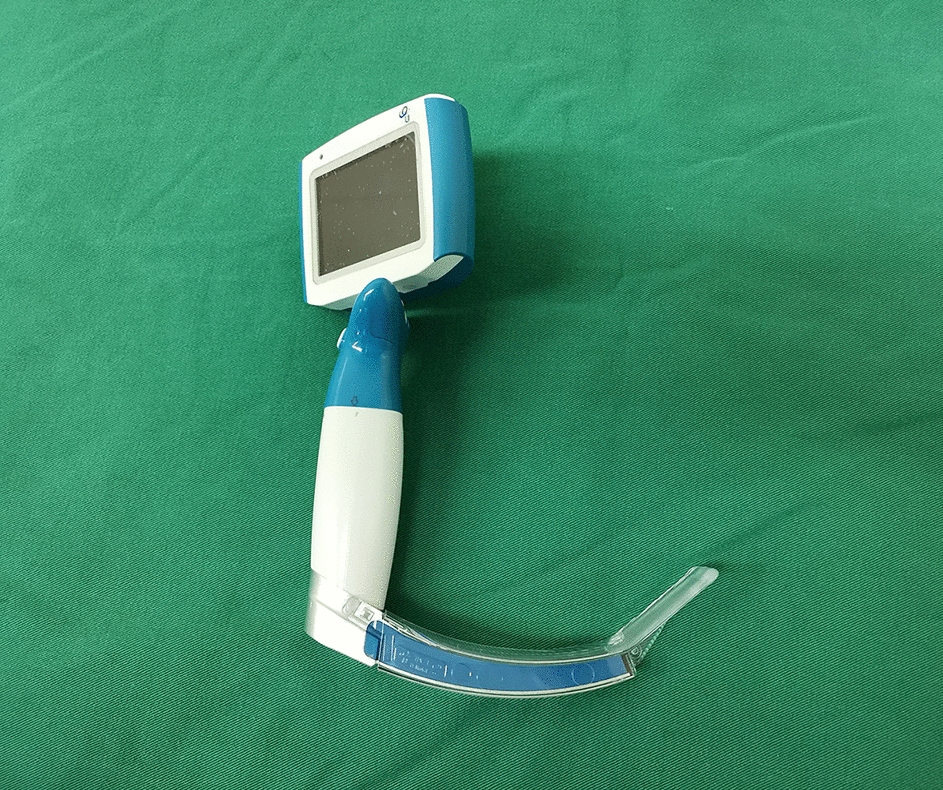
Video laryngoscope

All participants were informed in detail about the characteristics of the flexible video laryngoscope and how to operate it before practice. Before measurement, each participant performed intubation twice using the FVL and a video laryngoscope (TDC-C3, Zhejiang UE Medical Corp, Xianju City, China; Fig. 4) in the neutral position with intermediate and difficult mouth opening, respectively. The study was conducted immediately after practice, and each intubation was conducted only once. Tooth loss, esophageal intubation, and attempts requiring more than 90 s were recorded as intubation failure [19]. Participants performed intubation in the difficult airway management simulator in the neutral position with intermediate mouth opening first and then in the neutral position with difficult mouth opening. Before the study began, the sequence of laryngoscope use (flexible video laryngoscope first or video laryngoscope first) was randomly allocated by a random number table (Randomization Adviser 1.0, Beijing, China). A reinforced tube (7.0 mm ID, Covidien IIc, Athlone, Ireland) was selected and shaped by the participant before video laryngoscope intubation. The guiding tube was also installed with the ETT in advance before FVL intubation. The successful intubation, intubation time, and classification of glottic exposure were recorded. The difficulty of tracheal intubation of those two laryngoscopes was assessed by the participants using the numeric rating scale (NRS, Numerical Rating Scale of 1–10, from very easy to very difficult). Successful intubation referred to inserting the endotracheal tube into the trachea. The intubation time was defined as the time from insertion of the tip of the laryngoscope between the teeth until the endotracheal tube was inserted into the trachea. The intubation time was recorded as 90 s if intubation failure occurred. Glottic exposure was assessed by the Cormack Lehane score [20].

### Statistical analysis

The primary outcome was the success rate of intubation. The secondary outcomes were intubation time, classification of glottic exposure and difficulty score of intubations.

The rate of successful intubation was tested by the McNemar test. The comparison of intubation time, the difficulty score of intubations and the Cormack–Lehane score was compared by the Wilcoxon test. For all comparisons, a *P* value < 0.05 was considered to be statistically significant. Statistical analyses were performed with SPSS version 20.0 (SPSS Inc., Chicago, IL, USA).

According to the preliminary experiment in the neutral position with difficult mouth opening, based on the sample size estimation of the paired design, the success rate of endotracheal intubation with FVL was 100%, and the success rate of endotracheal intubation with video laryngoscopy was 33.3%. For a two-tailed comparison, with *β* = 0.1 and *a *= 0.05, a sample size of no less than 6 participants was needed.

## Supplementary Information

**Additional file 1.** The intubation video of the flexible video laryngoscope.

## Data Availability

The data sets used and/or analysed during the current study are available from the corresponding author on reasonable request.

## Abbreviations

FVL: Flexible video laryngoscope
ETT: Endotracheal tube
